# An Improved Adaptive Spatial Preprocessing Method for Remote Sensing Images

**DOI:** 10.3390/s21175684

**Published:** 2021-08-24

**Authors:** Liangliang Zheng, Wei Xu

**Affiliations:** 1 Changchun Institute of Optics, Fine Mechanics and Physics, Chinese Academy of Sciences, Changchun 130033, China; xuwei@ciomp.ac.cn; 2 Key Laboratory of Space-Based Dynamic & Rapid Optical Imaging Technology, Chinese Academy of Sciences, Changchun 130033, China

**Keywords:** image preprocessing, adaptive spatial filter, noise removal, edge sharpness

## Abstract

Since remote sensing images are one of the main sources for people to obtain required information, the quality of the image becomes particularly important. Nevertheless, noise often inevitably exists in the image, and the targets are usually blurred by the acquisition of the imaging system, resulting in the degradation of quality of the images. In this paper, a novel preprocessing algorithm is proposed to simultaneously smooth noise and to enhance the edges, which can improve the visual quality of remote sensing images. It consists of an improved adaptive spatial filter, which is a weighted filter integrating functions of both noise removal and edge sharpness. Its processing parameters are flexible and adjustable relative to different images. The experimental results confirm that the proposed method outperforms the existing spatial algorithms both visually and quantitatively. It can play an important role in the remote sensing field in order to achieve more information of interested targets.

## 1. Introduction

The remote sensing image is an important source for people to achieve a variety of useful information; thus, many airborne or aerospace imaging systems are developed to acquire high quality remote sensing images, which are based on photoelectric detectors with high sensitivity. The quality of the image becomes particularly important because it affects the accurate interpretation and perception of the image. Nevertheless, noise often inevitably exists in the image, and the targets are usually blurred to a certain extent due to the acquisition of the imaging system, resulting in the degradation of quality of the images [1,2,3,4]. Therefore, it makes it hard for human observers to discriminate the fine details of the images such as edges and other features.

There are many kinds of noises interfering the imaging system, principally from the photoelectric detectors, for example, photon shot noise, dark current noise, thermal noise, and so on. The images acquired by the system always include noise, which affects their final display effect [5,6,7]. Therefore, some image preprocessing techniques can be applied to obtain the image data of high definition and high signal-to-noise ratio (SNR) by denoising and edge sharpening. The high-quality images often possess more abundant information and higher value. The expected processing effect can not only smooth the noise in the uniform region to improve the SNR but also sharpen the edges in the target region to achieve clearer images [8,9]. Thus, the design of the preprocessing algorithm is particularly critical. It should not amplify the noise or obscure useful edge information.

Currently, there are many image preprocessing methods that can be generally divided into two kinds: space domain and transform domain. The objective of any filtering methods is to simultaneously remove noise and to retain the important features of the images. The methods based on space domain mainly include Wiener filter, Gauss filter, bilateral filter, neighborhood medium filter, average filter, and so on [5,10,11,12,13,14,15,16,17]. The image grayscale of each pixel is directly dealt with to achieve the desired effect. The transform domain methods usually consist of discrete wavelet transform (DWT), discrete cosine transform (DCT), and Fourier transform, etc. [5,11,18,19,20,21,22,23]. The image is first transferred into the frequency domain by transforming, and the processing operations are carried out. Then, the inverse transform is performed to obtain the resultant image. Some of these approaches are quite computationally intensive. By contrast, the former is easier to be implemented without any transforms. They can smooth the noise effectively, but most of them only preserve the edge information of the images and are unable to enhance it. The high frequency region of an image often plays a vital role in enhancing its visual appearance, with respect to the edges and contrast. The classic linear unsharp masking filter (UMF) is one of the popular sharpening techniques that is capable of magnifying the high-frequency content, but it is highly sensitive to the noise present in the original image [15,16,24,25,26]. Although an algorithm combining the bilateral filter and the UMF can sharpen the edges, presented in Reference [27], the resultant images are not satisfactory enough, producing overshoot and undershoot artifacts.

In this work, a novel spatial preprocessing algorithm is proposed, called an improved adaptive spatial filter (IASF). It has good capabilities of both edge sharpening and noise smoothing, and its processing parameters are flexible and adjustable relative to different images. It can enhance the edges in the target region without artifacts and smooth the noise in the uniform region without reducing the useful information. Therefore, it renders the IASF filter more appropriate for processing remote sensing images. Its performances in edge sharpening and denoising are analyzed and compared with the commonly used spatial algorithms. The experimental results indicate that the proposed method performs better in both visual effect and objective data. It can play an important role in the remote sensing field in making images clearer with higher SNR and better display effects.

## 2. Related Work

This section may be divided by subheadings. It should provide a concise and precise description of the experimental results, their interpretation, as well as the experimental conclusions that can be drawn.

In this section, we mainly discuss the spatial filtering techniques that are the most related to our proposed algorithm.

### 2.1. Wiener Filter

It is a classic approach based on statistics, which filters out the noise present in the image and preserves the images’ details [5,12]. Its filtering principle is state as follows:

(1) $$\hat{g}\left(i,j\right)=\frac{\sigma_{n}^{2}}{\sigma_{I}^{2}}\times \bar{g}\left(i,j\right)+\frac{\sigma_{I}^{2}-\sigma_{n}^{2}}{\sigma_{I}^{2}}\times g\left(i,j\right),\;$$

where g(i,j) is the intensity value of pixel (*i*,*j*) and $\bar{g}\left(i,j\right)$ is the average intensity of pixels in the *M*×*N* window centered at (*i*,*j*). $\sigma_{n}^{2}$ and $\sigma_{I}^{2}$ represent the variances of the noise and the actual image, respectively. Its performance can be analyzed in two cases.

Case 1: “Target region.” The variance $\sigma_{I}^{2}$ is far greater than $\sigma_{n}^{2}$, that is, $\sigma_{I}^{2}\gg \sigma_{n}^{2}$, and we can obtain $\frac{\sigma_{n}^{2}}{\sigma_{I}^{2}}\approx 0$ and $\frac{\sigma_{I}^{2}-\sigma_{n}^{2}}{\sigma_{I}^{2}}\approx 1$; thus, $\hat{g}\left(i,j\right)\approx g\left(i,j\right)$. This means that the filter can preserve the edge information of the image.

Case 2: “Uniform region.” The variance $\sigma_{I}^{2}$ approximates $\sigma_{n}^{2}$, that is, $\sigma_{I}^{2}\approx \sigma_{n}^{2}$, and we can obtain $\frac{\sigma_{n}^{2}}{\sigma_{I}^{2}}\approx 1$ and $\frac{\sigma_{I}^{2}-\sigma_{n}^{2}}{\sigma_{I}^{2}}\approx 0$; thus, $\hat{g}\left(i,j\right)\approx \bar{g}\left(i,j\right)$. This means that the filter becomes an average filter in order to smooth the noise of the image.

Therefore, it can be concluded that the Wiener filter is an edge-preserving smoothing filter with advantages of self-adaptation and easy-implementation. However, it only tries to preserve the edges instead of enhancing them.

### 2.2. Bilateral Filter

The de-noising bilateral filter is built based on the low-pass Gaussian algorithm, which considers both distance between the pixels and the intensity variations of the image [10,16]. That is the domain filter and the range filter. Its working principle can be expressed as follows:

(2) $$\hat{g}\left(i,j\right)=\underset{k}{\sum }\underset{l}{\sum }h\left(i,j;k,l\right)g\left(k,l\right),$$

where $\hat{g}\left(i,j\right)$ is the restored image and h(i,j;k,l) is the response at (*i*,*j*) to an impulse at (*k*,*l*) [10]. The specific definition of h(i,j;k,l) can be expressed as follows:

(3) $$h\left(i_{0},j_{0};i,j\right)\;=\;\{\begin{array}{c} r_{i_{0},j_{0}}^{-1}exp\left(-\frac{{\left(i-i_{0}\right)}^{2}+{\left(j-j_{0}\right)}^{2}}{2\sigma_{d}^{2}}\right)exp\left(-\frac{{\left(g\left(i,j\right)-g\left(i_{0},j_{0}\right)\right)}^{2}}{2\sigma_{r}^{2}}\right),\\ \left(i,j\right)\in \Omega_{i_{0},j_{0}}\\ 0,\;\mathrm{else} \end{array}\;,\;\;$$

where ($i_{0},j_{0}$) is the central pixel of the window,$\;\Omega_{i_{0},j_{0}}=\left\{\left(i,j\right):\left(i,j\right)\in \left(i_{0}-M,i_{0}+M\right)\times \left(j_{0}-N,j_{0}+N\right)\right\}$; $\sigma_{d}$ and $\sigma_{r}$ are the standard deviations of the domain and range Gaussian filters, respectively; and $r_{i_{0},j_{0}}$ is the normalization factor that makes the average intensity of the whole image unchanged. Its definition is given by the following.

(4) $$r_{i_{0},j_{0}}=\overset{i_{0}+M}{\underset{i=i_{0}-M}{\sum }}\overset{j_{0}+N}{\underset{j=j_{0}-N}{\sum }}exp\left(-\frac{{\left(i-i_{0}\right)}^{2}+{\left(j-j_{0}\right)}^{2}}{2\sigma_{d}^{2}}\right)exp\left(-\frac{{\left(g\left(i,j\right)-g\left(i_{0},j_{0}\right)\right)}^{2}}{2\sigma_{r}^{2}}\right).\;$$

The domain low-pass Gaussian filter can provide higher weight to pixels that are spatially close to the central pixel. The range low-pass Gaussian filter can provide higher weight to pixels that are similar to the central pixel in intensity. Thus, the performance of the bilateral filter mainly depends on two parameters, $\sigma_{d}$ and $\sigma_{r}$, when the size of the window is fixed.

Zhang B et al. focused on the characteristics of the range filter, increasing a variable *ε* to sharpen the edge information [10]. Then, the weighted factor of the range filter becomes the following:

(5) $$W_{r}^{'}=exp\left(-\frac{{\left(g\left(i,j\right)-g\left(i_{0},j_{0}\right)-\varepsilon \left(i_{0},j_{0}\right)\right)}^{2}}{2\sigma_{r}^{2}}\right),\;\;\;\;\;\;\;\;\;$$

where $\varepsilon \left(i_{0},j_{0}\right)$ represents the grayscale offset of the center pixel $\left(i_{0},j_{0}\right)$. When $\varepsilon \left(i_{0},j_{0}\right)=0$, $W_{r}^{\text{'}}$ is the conventional weighted factor of the range filter. If g(i,j) is similar to the center pixel $g\left(i_{0},j_{0}\right)$, its weighted factor will be larger. Therefore, this filter produces the result that the grayscale of the pixel approaches $g\left(i_{0},j_{0}\right)$ to preserve the edges. When $\varepsilon \left(i_{0},j_{0}\right)\neq 0$, the filter will provide higher weight to pixels that are similar to $\left(g\left(i_{0},j_{0}\right)+\varepsilon \left(i_{0},j_{0}\right)\right)$. Thus, this filter makes the grayscale of the pixel approach $\left(g\left(i_{0},j_{0}\right)+\varepsilon \left(i_{0},j_{0}\right)\right)$. It can be observed that this filtering algorithm can enhance the edges, provided a reasonable $\varepsilon \left(i_{0},j_{0}\right)$ is selected.

### 2.3. UMF Filter

Unsharp mask filter (UMF), a high-pass linear filtering method, is a typical edge enhancing algorithm with very low-cost computational structure [24,25,26]. The neighborhood of the filtering operator is a fixed 3 × 3 matrix, as shown in Equation (6), where υ determines the direction of the edge sharpening and belongs to the interval [0, 1]. If υ=0, the filter will sharpen the image in the horizontal and vertical directions, respectively. If υ=1, the sharpening direction changes to both diagonals. If 0<υ<1, the edges will be sharpened in a superimposed direction. Although UMF can enhance the edge information effectively, it also amplifies the noise in the image.

(6) $$\kappa =\frac{1}{\upsilon +1}\left[\begin{array}{ccc} -\upsilon & \upsilon -1 & -\upsilon \\ \upsilon -1 & \upsilon +5 & \upsilon -1\\ -\upsilon & \upsilon -1 & -\upsilon \end{array}\right].$$

## 3. Improved Adaptive Spatial Filter (IASF) for Image Sharpening and Denoising

A weighted normalization filtering algorithm was proposed for suppressing the noise efficiently in order to further enhance the SNR and sharpen the main edge information simultaneously. It introduces the improved range filter and combines the average filter, called the improved adaptive spatial filter (IASF). The principle is as follows:

(7) $$\hat{g}\left(i,j\right)=a\times g_{r}\left(i,j\right)+b\times \bar{g}\left(i,j\right),\;\;\;$$

where $g_{r}\left(i,j\right)$ represents the output of the improved range filter. The parameters *a* and *b* are the normalized weighted factors of the improved range filter and the average filter, respectively, rendering the filter adaptable to different image data.

Therefore, the proposed method has the advantages of both filters mentioned above. The average filter can smooth the noise effectively, and the improved range filter can sharpen the edges of the image. Consequently, the IASF filter integrates the abilities of both noise suppressing and edge sharpening, which meets the processing need of remote sensing image data.

The performance of the proposed algorithm is closely related to the design of the improved range filter and the reasonable selections of weighted factors *a* and *b*. These will be analyzed and discussed in the following part.

### 3.1. Design of the Improved Range Filter

**Figure 1:**
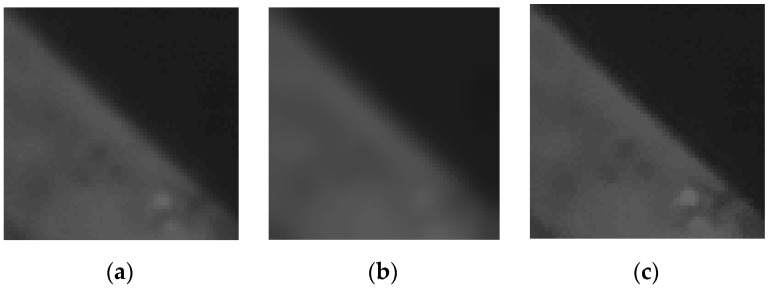
The different results of the improved range filter with different grayscale offsets. (**a**) Original image; (**b**)$\;\varepsilon \left(i_{0},j_{0}\right)=MEAN-g\left(i_{0},j_{0}\right)$; (**c**)$\;\varepsilon \left(i_{0},j_{0}\right)=g\left(i_{0},j_{0}\right)-MEAN$.

It is necessary to select a reasonable grayscale offset $\varepsilon \left(i_{0},j_{0}\right)$ for each pixel in order to enhance the edge information, which will be analyzed in the following two cases. There is a mean grayscale value, denoted as *MEAN*, in the neighborhood $\Omega_{i_{0},j_{0}}$.

(a) $\varepsilon \left(i_{0},j_{0}\right)=MEAN-g\left(i_{0},j_{0}\right)$. The pixel $g\left(i_{0},j_{0}\right)$ moves to *MEAN*, resulting in a blurred image, because each pixel tends to the average value of its neighborhood in the image, as shown in Figure 1b.

(b) $\varepsilon \left(i_{0},j_{0}\right)=g\left(i_{0},j_{0}\right)-MEAN$. The pixel $g\left(i_{0},j_{0}\right)$ moves away from *MEAN*, making each pixel tend to $\left(g\left(i_{0},j_{0}\right)+\varepsilon \left(i_{0},j_{0}\right)\right)$, resulting in the sharpening effect, as shown in Figure 1c. It can be divided into three cases. If $g\left(i_{0},j_{0}\right)<MEAN$, that is $\varepsilon \left(i_{0},j_{0}\right)<0$, the grayscale of the pixel decreases. Otherwise, if $g\left(i_{0},j_{0}\right)>MEAN$, $\varepsilon \left(i_{0},j_{0}\right)>0$, the grayscale of the pixel increases. Otherwise, if $g\left(i_{0},j_{0}\right)=MEAN$, $\varepsilon \left(i_{0},j_{0}\right)=0$, it becomes a conventional range filter.

According to the above analysis, it can be observed that the improved range filter can reduce the transition pixels of the edges effectively and increase the gradient of the grayscale variation in order to achieve the edge enhancement when the pixel $g\left(i_{0},j_{0}\right)$ moves away from the *MEAN*. Now the proposed method sets $\varepsilon \left(i_{0},j_{0}\right)=g\left(i_{0},j_{0}\right)-MEAN$ as the grayscale offset in order to sharpen edges.

### 3.2. Determination of the Weighted Factors of IASF

The weighted factors *a* and *b* are a set of certain values in each window $\Omega_{i_{0},j_{0}}$. In order to determine the reasonable weighted factors, a cost function is employed to minimize the difference between $\hat{g}\left(i,j\right)$ and g(i,j) in window $\Omega_{i_{0},j_{0}}$:

(8) $$\left\{a^{*},b^{*}\right\}=arg\;\underset{\left\{a,b\right\}}{min}\underset{\left(i,j\right)\in \Omega_{i_{0},j_{0}}}{\sum }\left({\left(a\times g_{r}\left(i,j\right)+b\times \bar{g}\left(i,j\right)-g\left(i,j\right)\right)}^{2}+\epsilon a^{2}\right),\;\;$$

where ϵ is a regularization parameter and a positive real number. Its specific meaning is discussed in the following part. Equation (8) is a linear ridge regression model, and its solution can be expressed as follows.

(9) $$\{\begin{array}{c} a^{*}=\frac{\sigma_{I}^{2}}{\sigma_{I}^{2}+\epsilon }\\ b^{*}=1-a^{*}=\frac{\epsilon }{\sigma_{I}^{2}+\epsilon } \end{array}\;\;\;\;.\;\;\;\;\;\;$$

Therefore, taking Equation (9) into Equation (7), we can obtain the following equation.

(10) $$\hat{g}\left(i,j\right)=\frac{\sigma_{I}^{2}}{\sigma_{I}^{2}+\epsilon }\times g_{r}\left(i,j\right)+\frac{\epsilon }{\sigma_{I}^{2}+\epsilon }\times \bar{g}\left(i,j\right).\;\;$$

The performance of the IASF can be analyzed in two typical cases based on Equation (10).

Case 1: “Edge region.” The image data changes a lot within $\Omega_{i_{0},j_{0}}$, so the variance $\sigma_{I}^{2}$ is far larger than ϵ, $\sigma_{I}^{2}\gg \epsilon$. Thus, we can obtain the weighted factor $\frac{\sigma_{I}^{2}}{\sigma_{I}^{2}+\epsilon }\approx 1$ and $\frac{\epsilon }{\sigma_{I}^{2}+\epsilon }\approx 0$, resulting in $\hat{g}\left(i,j\right)\approx g_{r}\left(i,j\right)$. This makes the IASF sharpen the edge information.

Case 2: “Uniform region.” The image variance $\sigma_{I}^{2}$ is far less than ϵ, $\sigma_{I}^{2}\ll \epsilon$. Thus, we can obtain the weighted factor $\frac{\sigma_{I}^{2}}{\sigma_{I}^{2}+\epsilon }\approx 0$ and $\frac{\epsilon }{\sigma_{I}^{2}+\epsilon }\approx 1$, resulting in $\hat{g}\left(i,j\right)\approx \bar{g}\left(i,j\right)$. The IASF turns out to be an average filter for smoothing the noise.

In total, the weighted factors of the IASF can adjust itself to implement the edge sharpening and noise suppressing simultaneously according to different images. More specifically, the criterion of an “edge region” or a “uniform region” is determined by the parameter ϵ. The regions with variance $\sigma_{I}^{2}$ far less than ϵ are smoothed, whereas those with variance much larger than ϵ are enhanced. The effect of parameter ϵ in the IASF is similar to the variance $\sigma_{r}^{2}$ in the range filter, both of which can determine whether an edge region should be enhanced or preserved. Thus, both parameters are equivalent, so we empirically set $\epsilon =\sigma_{r}^{2}$ [13].

In order to further improve the display quality of the remote sensing images and to enhance the edge sharpening of the image information, the grayscale offset is set to $\varepsilon \left(i_{0},j_{0}\right)=k_{p}\times \left(g\left(i_{0},j_{0}\right)-MEAN\right)$ in this method. Thus, the weighted factor of the improved range filter becomes the following.

(11) $$W_{r}^{'}=exp\left(-\frac{{\left(g\left(i,j\right)-g\left(i_{0},j_{0}\right)-k_{p}\times \left(g\left(i_{0},j_{0}\right)-MEAN\right)\right)}^{2}}{2\sigma_{r}^{2}}\right).\;\;\;$$

At the same time, in order to improve the adaptability of the algorithm, we introduced the rational parameter *k_r_* to optimize the functions of edge sharpening and denoising for different images. Thus, the filtering algorithm can be expressed as follows.

(12) $$\hat{g}\left(i,j\right)=\frac{\sigma_{I}^{2}}{\sigma_{I}^{2}+k_{r}\sigma_{r}^{2}}\times g_{r}\left(i,j\right)+\frac{k_{r}\sigma_{r}^{2}}{\sigma_{I}^{2}+k_{r}\sigma_{r}^{2}}\times \bar{g}\left(i,j\right).\;\;\;\;\;\;\;\;\;\;\;\;\;\;\;\;\;\;\;\;\;\;\;\;\;\;\;\;\;\;\;\;\;\;$$

The function and selection of parameters *k_p_* and *k_r_* are described below.

### 3.3. Parameter k_p_

The parameter *k_p_* is an adjusting factor of the grayscale offset for enhancing edge sharpening. The higher the value is, the larger the grayscale offset, producing a more obvious sharpening effect. However, if the parameter *k_p_* is too large, it results in grayscale overshoot. Thus, *k_p_* should not be too large. Generally, the range of *k_p_* is 1 ≤ *k_p_* ≤ 2, which can be adjusted according to the actual image data. The parameter *k_p_* is set to 1.5 by training the actual images here.

### 3.4. Parameter k_r_

The parameter *k_r_* is a weighted factor for measuring the effect of noise smoothing. The smaller the value is, the weaker the smoothing effect and the stronger the sharpening effect and vice versa. Now, we set two different thresholds *a* and *b* to divide the parameter *k_r_* into three segments. In order to simplify the computation, we employ three linear expressions to represent *k_r_* here. When $\sigma_{I}^{2}<a^{2}$, *k_r_* is set to 1, indicating the edge sharpening and noise smoothing based on the actual image. When $\sigma_{I}^{2}>b^{2}$, *k_r_* is set to 0.01, showing that the neighborhood is mainly the edge region and performing stronger edge sharpening. In the other cases, the parameter *k_r_* can be expressed as a linear expression. Consequently, we have the following.

(13) $$k_{r}\;=\;\{\begin{array}{l} 1,\;if\;\sigma_{I}^{2}<a^{2}\;\\ 0.01,\;if\;\sigma_{I}^{2}>b^{2}\\ 1-\frac{0.99}{b^{2}-a^{2}}\times \left(\;\sigma_{I}^{2}-a^{2}\right),\;\;if\;a^{2}\leq \sigma_{I}^{2}\leq b^{2}\; \end{array}.\;$$

The parameters *a* and *b* are selected in terms of the variance of images with different features. We will set *a* = 1.5 and *b* = 8 by training the actual images here.

## 4. Verification and Discussion

**Figure 2:**
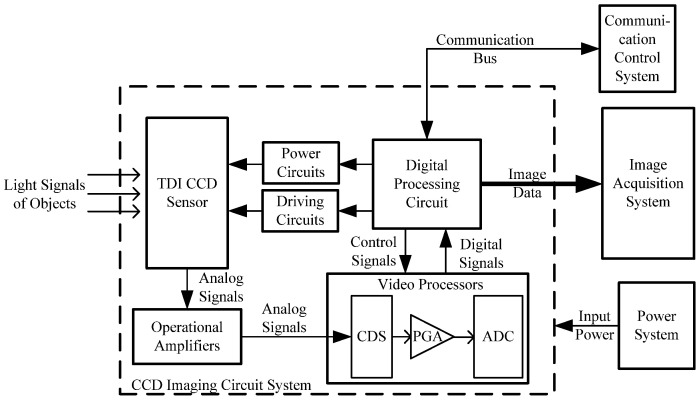
Test block diagram of the TDI CCD imaging system.

The test images are acquired by a time-delayed integration (TDI) charge-coupled device (CCD) imaging system, which employs a kind of linear array photoelectric detector. It can implement charge accumulations by superposition mode with advantages of high sensitivity, high dynamic range, and low noise. The imaging system is usually composed of TDI CCD sensor, amplifiers, video processors, digital processing circuit, and so on, represented in Figure 2. It can realize the driving of TDI CCD, the power supply, signal quantization, and output of the image data. The communication control system is in charge of supervising and monitoring the imaging system. The image data can be captured and displayed by the image acquisition system. The main technical indicators of the imaging system are as shown in Table 1.

Then, we apply the imaging system in order to acquire multiple images with different targets and choose two of them with size of 256 pixels × 256 pixels to validate the performance of the proposed method. Various spatial filters have been applied to the test images for comparison.

Some performance indexes are applied for the quantitative evaluation in order to compare the processing effects. There are two commonly used objective indexes: the mean value *μ* and standard deviation (STD) *σ* [5,11]. Their definitions are given by the following:

(14) $$\mu =\frac{1}{MN}\overset{M}{\underset{i=1}{\sum }}\overset{N}{\underset{j=1}{\sum }}g\left(i,j\right),$$

(15) $$\sigma =\sqrt{\frac{1}{MN}\overset{M}{\underset{i=1}{\sum }}\overset{N}{\underset{j=1}{\sum }}{\left(g\left(i,j\right)-\mu \right)}^{2}},\;$$

where g(i,j) is the intensity of pixel (*i*, *j*) and (*M*,*N*) is the size of the image region. The mean value *μ* is the average of all intensities, indicating the average brightness of the image. The STD(*σ*) is the deviation of intensities relative to the mean value, denoting the distribution uniformity of the pixels.

The gray mean gradient (GMG) is employed for measuring the edge sharpening effect, which is an image quality evaluation method based on the image gradient. Its expression is given by the following.

(16) $$\mathrm{GMG}=\overset{M-1}{\underset{i=1}{\sum }}\overset{N-1}{\underset{j=1}{\sum }}\frac{{\left(g\left(i+1,j\right)-g\left(i,j\right)\right)}^{2}+{\left(g\left(i,j+1\right)-g\left(i,j\right)\right)}^{2}}{2}\;\;\;.\;$$

The GMG indicates the variance rate of the grayscale of the image, representing the sharpness extent with higher sensitivity. The greater the value of GMG, the better the image quality; that is, the edges become sharper with more information of the targets. However, if there is a lot of noise in the image, the GMG may be bigger as well because both the noise and the edge are presented as high frequency component. Therefore, we apply the evaluation method by combining subjective visual effects and objective data comparisons in order to analyze the resultant images.

The size of the neighborhood window should be appropriate and selective. If it is too small, it cannot cover most of the edge transitions. Otherwise, if it is too big, it might increase the computation and consume time. Considering the above factors, we chose a 7 × 7 window. The standard deviation *σ_r_* of the IASF determines how selective the filter is in choosing the pixels that are similar enough in intensity, and *σ_r_* is set to two here.

**Figure 3:**
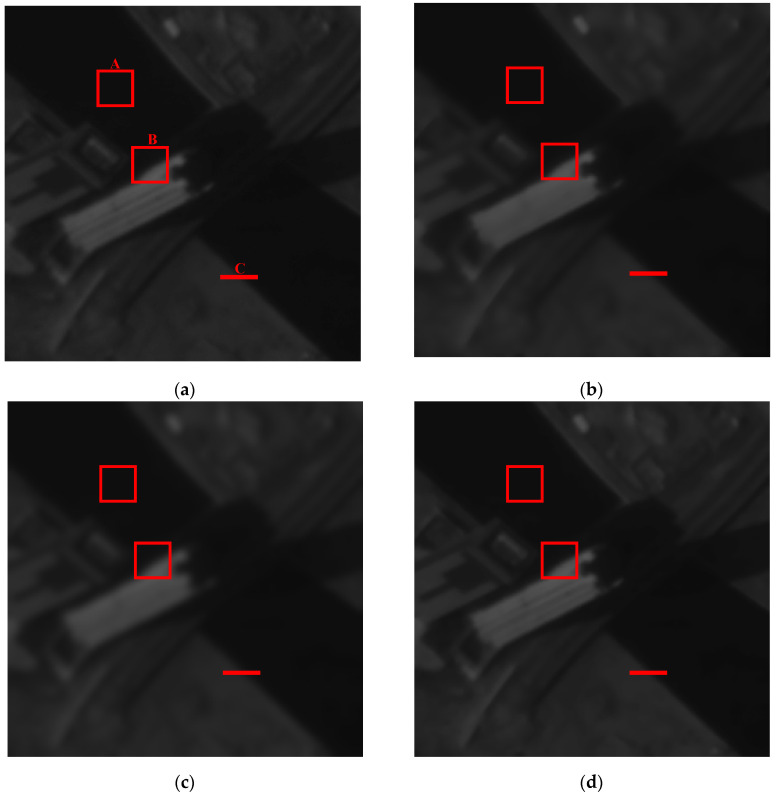

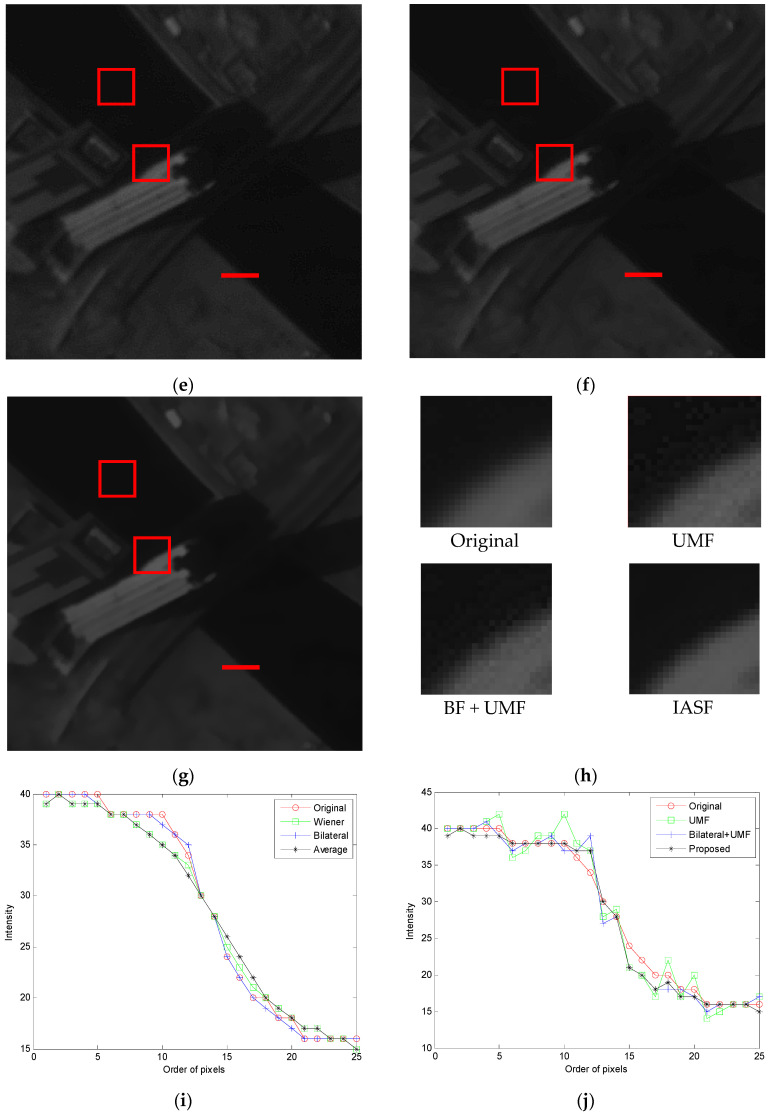
The raw image data of target 1 and its processed resultant images by different filters: (**a**) original data; (**b**) Wiener filter (WF); (**c**) average filter (AF); (**d**) bilateral filter(BF); (**e**) UMF; (**f**) BF + UMF; (**g**) IASF; (**h**) zoomed in images of box B; (**i**) gray level distributions of edge C for (**a**–**d**); (**j**) gray level distributions of edge C for (**e**–**g**).

Figure 3a is the raw image data of target 1 “building”, which is processed by different spatial filters as shown in Figure 3b–g. Box A shows a uniform region while box B is an edge region. The zoomed in images of box B for UMF, BF + UMF, and IASF are shown in Figure 3h. The different gray level distributions of edge C are depicted in Figure 3i,j.

Compared with the raw image Figure 3a, Figure 3b–d became blurred, and some details are lost from visual effects. From Figure 3i, it can be observed that the average filter (AF) makes the image the most blurred, increases the transition pixels, and reduces the slope of grayscale variation. The Wiener filter (WF) obtains similar results relative to the average filter, and the results of the bilateral filter (BF) becomes a little blurred, which is similar to the original. We can observe that the edge sharpening effect is realized in Figure 3e–g, but in terms of Figure 3h, the UMF obviously amplifies the noise in the image, resulting in visually less pleasing enhanced effects. For the combined algorithm of BF + UMF, it achieves a slightly better result than UMF due to the noise suppression by BF, but there is still some noise remaining in the image. However, the IASF implements the edge sharpening without noise amplification, producing a better visual effect, as shown in Figure 3g,h.

The grayscale variations of the three filters for edge C are described in Figure 3j. The UMF, sensitive to the noise, produces large overshoot and undershoot in grayscale, causing the undesired artifacts, although the slope of the edge increases. The BF + UMF method can restrain the overshoot to a certain extent, but the processed result is still unsatisfactory. Distinct from UMF, the IASF is not sensitive to the noise, and it can adjust the grayscale of edges in the spatial domain to increase the slope without generating overshoot. Thus, the IASF can enhance the edge information effectively to improve the overall appearance of the image.

Then, we can analyze the image data of target 1 processed by different algorithms to realize the objective data comparison, as shown in Table 2. The average intensity of each filter is nearly the same as the original. The STDs for the red box A and B are calculated, representing the uniform region and the edge region, respectively, with the size of 25 pixels × 25 pixels, and the GMGs for box B are also displayed.

It can be observed that the WF, the AF, and the IASF have the same STD results when processing the uniform region A, which is better than the BF. Therefore, this indicates that IASF has good capability for noise smoothing. From the calculation results of GMG for the edge region B, we can observe that the WF, BF, and AF have smaller values than the original image, indicating that they have less information of the targets and only perform edge preservation, while the UMF, BF + UMF and IASF performs larger edge preservation. Thus, the three filters can enhance the edges in order to obtain more information. We can draw the same conclusion from the STD results. However, the UMF and BF + UMF also amplifies the noise at the same time, resulting in a less pleasing appearance. Since IASF is not sensitive to noise, it can achieve an ideal enhancing effect without artifacts. Consequently, the IASF can simultaneously achieve good performance in edge sharpening and noise smoothing. The filtering characteristics of each algorithm are summarized in Table 2.

**Figure 4:**
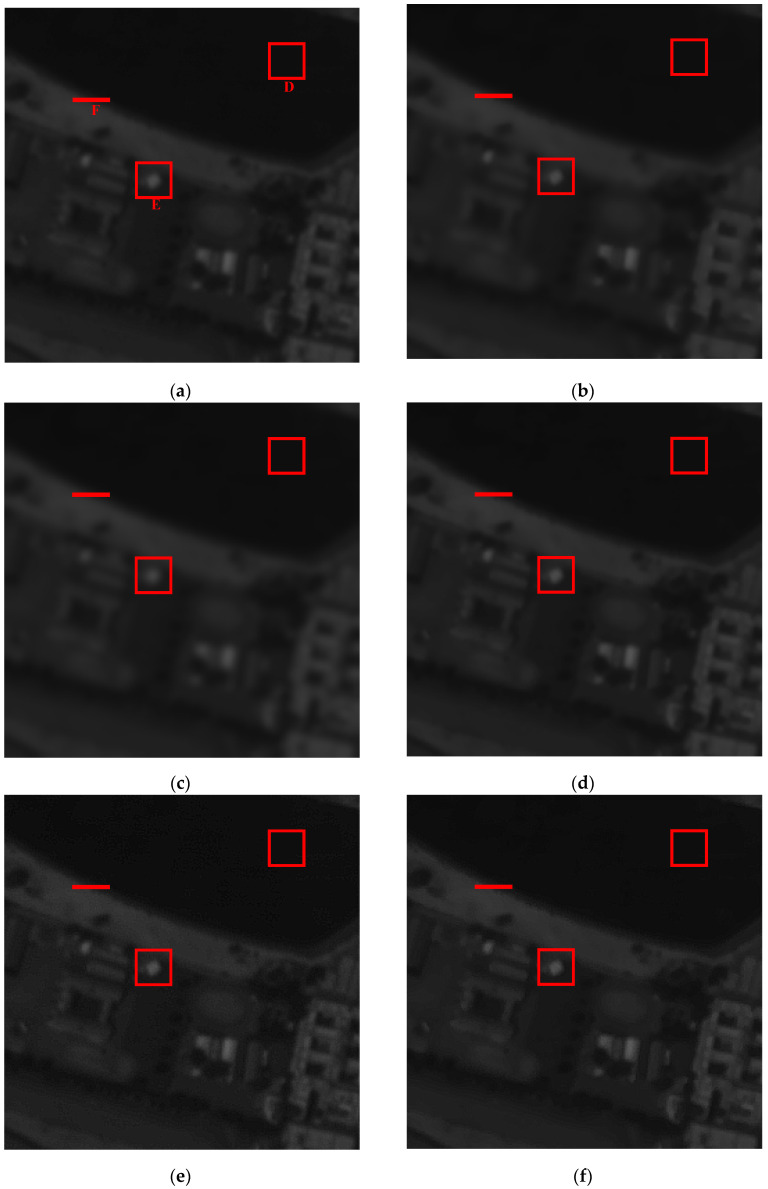

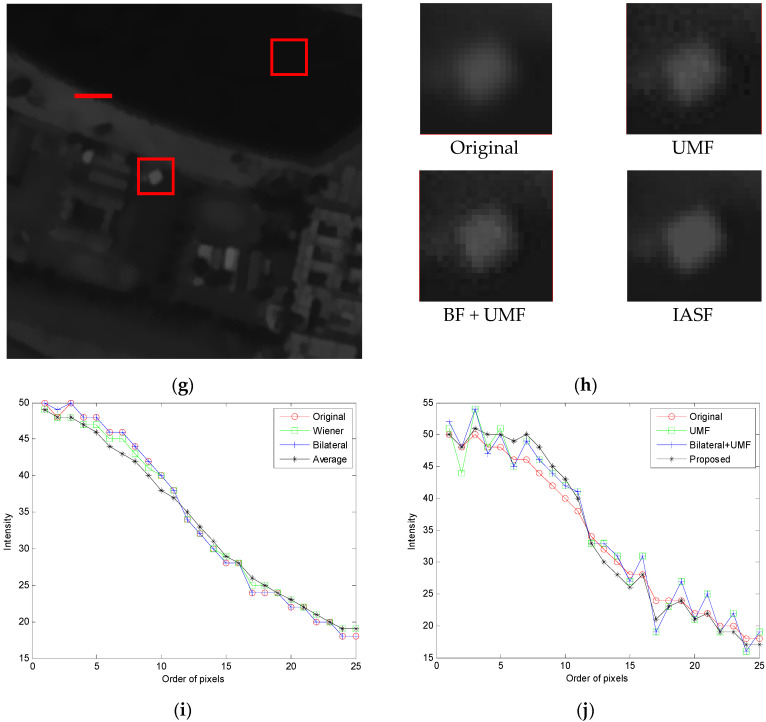
The raw image data of target 2 and its processed resultant images by different filters: (**a**) original data; (**b**) WF; (**c**) AF; (**d**) BF; (**e**) UMF; (**f**) BF + UMF; (**g**) IASF; (**h**) zoomed in images of box B; (**i**) gray level distributions of edge C for (**a**–**d**); (**j**) gray level distributions of edge C for (**e**–**g**).

We then change to another image to further validate the proposed method. Figure 4a is the raw image data of target 2 “beach” and its resultant processed images by different spatial filters, as shown in (b) to (g). Box D shows a uniform region while box E is an edge region. The zoomed in images of box E for UMF, BF + UMF, and IASF are shown in Figure 4h. The gray level distributions of edge F are depicted in Figure 4i,j.

It can be observed that, compared with the raw image Figure 4a, Figure 4b–d become blurred while Figure 4e–g become clearer with their edges enhanced. In terms of Figure 4h, since UMF is sensitive to the noise, it has a poor visual appearance. The BF + UMF obtained a slightly better result than UMF, but some noise still remained in the image. The IASF sharpens the edge information effectively without obvious noise, achieving a better visual appearance. From Figure 4i, we know that AF has the lowest slope of grayscale variation, resulting in the most blurred image. WF obtained a similar result relative to AF, while BF obtained a little blurred image, similar to the original.

The grayscale variations of the three filters for edge F are depicted in Figure 4j. Similarly to Figure 3j, the UMF produces large overshoot and undershoot, resulting in the undesired artifacts. Although the overshoot can be suppressed to a certain extent by BF, the BF + UMF method still cannot obtain a more pleasing result. Nevertheless, the IASF can adjust the grayscale of edges in spatial domain to increase the slope without producing artifacts through the reasonable selection of parameters *k_p_* and *k_r_*. Thus, the IASF can sharpen the edges effectively to improve the overall appearance of the image.

The objective data comparison results for the image data of target 2 are shown in Table 3. The average intensity of each filter is nearly the same as the original one. The STDs for the red boxes D and E are calculated, each with a size of 25 pixels × 25 pixels, and the GMGs for box E are also displayed.

It can be observed that IASF has nearly the same STD result with WF and AF when processing the uniform region D, better than the BF. Thus, the IASF has a good capability for noise smoothing. From the calculation results of GMG and STD for the edge region E, we can observe that the UMF, BF + UMF, and IASF have higher values than the original one, indicating that the three filters can enhance the edges to obtain more information. However, the UMF and BF + UMF also amplify the noise, resulting in less pleasing effects. The IASF can achieve an ideal enhancing effect without artifacts since it is not sensitive to noise.

**Figure 5:**
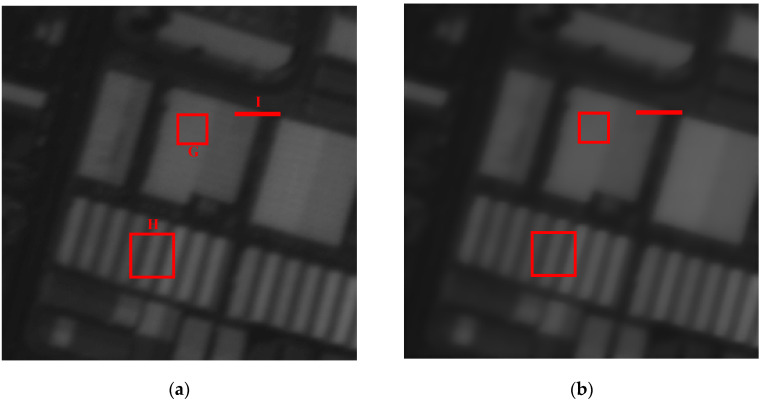

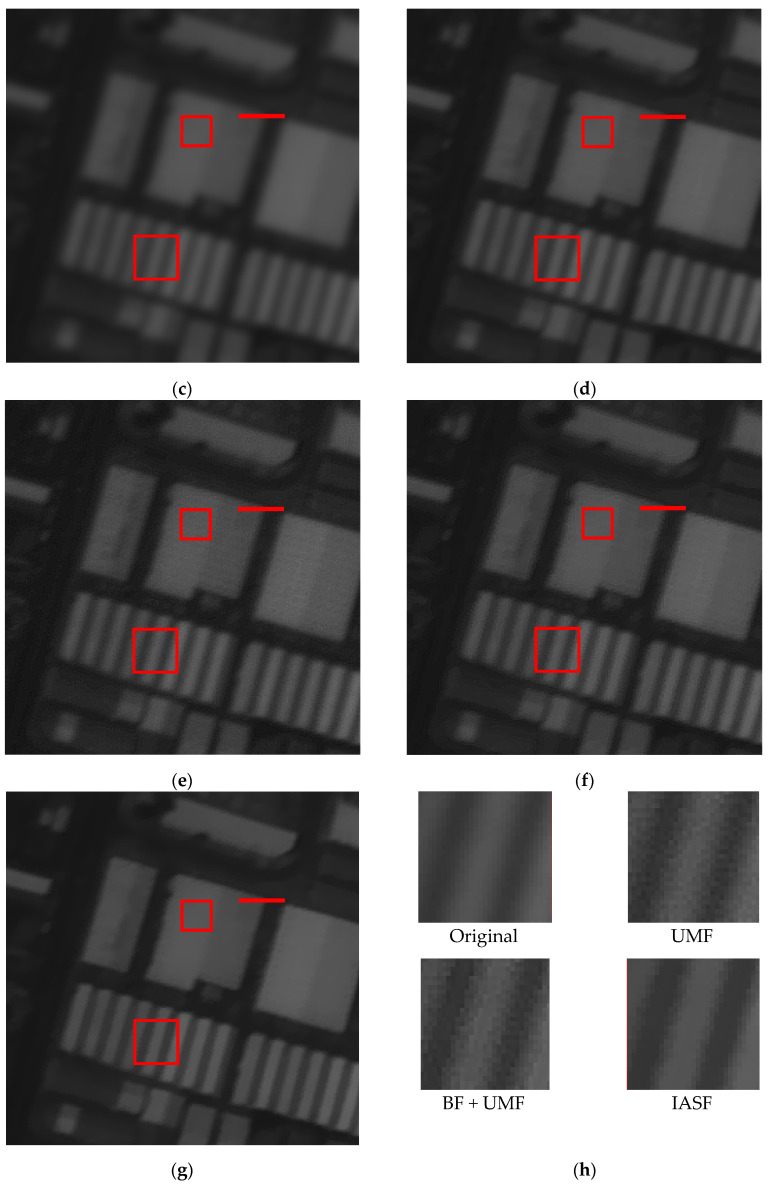

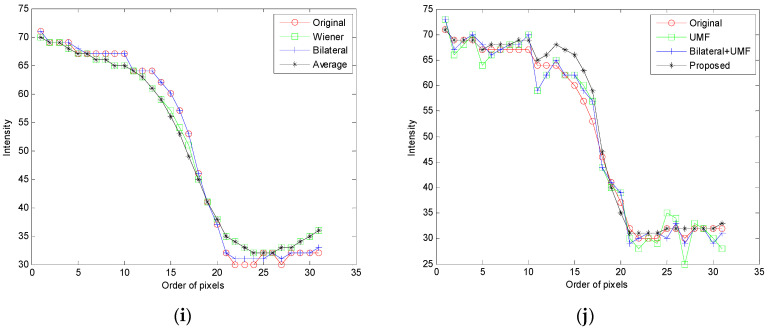
The raw image data of target 3 and its processed resultant images by different filters: (**a**) original data; (**b**) WF; (**c**) AF; (**d**) BF; (**e**) UMF; (**f**) BF + UMF; (**g**) IASF; (**h**) zoomed in images of box H; (**i**) gray level distributions of edge I for (**a**–**d**); (**j**) gray level distributions of edge I for (**e**–**g**).

Figure 5a is the raw image data of target 3 “town” and its processed resultant images by different spatial filters, as shown in (b) to (g). The box G shows a uniform region while the box H is an edge region. The zoomed in images of the box H for UMF, BF + UMF, and IASF are shown in Figure 5h. The gray level distributions of edge I are depicted in Figure 5i,j.

A similar conclusion can be drawn that, compared with the raw image Figure 5a, Figure 5b–d become blurred while Figure 5e–g become clearer with their edges enhanced. The UMF and the BF + UMF are sensitive to noise, which produces large overshoot and undershoot, resulting in poor visual appearances. However, the IASF can adjust the grayscale of edges in the spatial domain in order to sharpen edge information effectively without obvious noise, achieving better visual appearance.

The objective data comparison results for the image data of target 3 are shown in Table 4. It can be observed that IASF has nearly the same STD result with WF and AF when processing the uniform region G and is better than the BF, indicating a good capability for noise smoothing. According to the calculation results of GMG and STD for edge region H, we can observe that UMF, BF + UMF, and IASF can enhance the edge information with higher value. The IASF can achieve an ideal enhancing effect while UMF and BF + UMF simultaneously amplify noise. Consequently, the IASF can simultaneously achieve good performance in edge sharpening and noise smoothing.

The information entropy of image data is employed for evaluating the effect of edge sharpening, as shown in Table 5. We can observe that the information entropy results of the three filters increase in different degrees. However, UMF and BF + UMF not only sharpen the edges but also amplify the noise, resulting in higher values. The IASF is not sensitive to noise; thus, it can achieve better enhancing results.

In summary, the performance of the proposed preprocessing algorithm is verified and analyzed based on three different target images, and its related properties are described quantitatively from Table 2 to Table 5. We can perceive that the IASF has excellent capabilities in both edge sharpening and noise smoothing.

The two parameters *k_p_* and *k_r_* are key factors affecting the filtering performance. The parameter *k_p_* determines the output of $g_{r}\left(i,j\right)$ in formula (12), which can sharpen the edges of the image. If *k_p_* is too big (that is, the grayscale offset is too large), the IASF may result in a drastic sharpening effect, and the image appears over-sharpened. If *k_p_* is smaller than 1, the sharpening effect is weakened, unable to meet the preprocessing purpose. The parameter *k_r_* is complementary with *k_p_*, which determines the normalized weighted factors of $g_{r}\left(i,j\right)$ and $\bar{g}\left(i,j\right)$. If we increase *k_r_*, the weight of $g_{r}\left(i,j\right)$ decreases and that of $\bar{g}\left(i,j\right)$ increases, indicating that the sharpening effect is weaker and the smoothing effect is stronger. Therefore, we should select reasonable parameters for *k_p_* and *k_r_* in order to achieve the desired results according to the specific image data.

The IASF is not sensitive to noise. It can adjust the grayscale of the local pixels for transforming the histogram of the image. If we set *k_p_* = 0, the improved range filter changes to a conventional range filter. Then, the IASF becomes a low-pass filtering algorithm due to the range filter, and the average filter possesses low-pass characteristics. In particular, if we further set *k_r_* = 0, the proposed algorithm eventually turns out to be the range filter.

The value ranges of *k_p_* and *k_r_* are greater than or equal to 0. The filtering effect will be different according to different parameter values, and it is summarized in Table 6.

## 5. Conclusions

According to the preprocessing requirements of the remote sensing images, a novel spatial algorithm is proposed for simultaneously smoothing noise and for enhancing the edges. The weighted normalization filtering algorithm integrates the improved range filter and the average filter, and its processing parameters are flexible and adjustable relative to different images. We compared our algorithm with other commonly used spatial filters such as the Wiener filter, bilateral filter, average filter, UMF, and the combination of bilateral filter followed by UMF. The experimental results clearly indicate that our algorithm performs better than the filters mentioned previously, both in terms of subjective as well as quantitative analysis. The proposed algorithm exhibits excellent performances of both edge sharpness and noise removal. It can play an important role in the remote sensing field for achieving clearer images with higher SNR.

## Figures and Tables

**Table 1:** The main technical indicators of the imaging system.

Items	Specifications
Spectral Range	450 nm–800 nm
Pixel Size	8.75 μm × 8.75 μm
Spatial Pixels	4096
PGA (Programmable Gain Amplifier)	0 dB–36 dB

**Table 2:** The comparison results for image data of target 1.

Objects	Average Intensity	STD		GMG	Characteristics
		Box A	Box B	Box B	
Original data	27.380	0.2962	27.2403	404	-
WF	27.342	0.1192	26.4826	178	Edge preserving and noise smoothing
BF	27.373	0.1316	27.2237	290	Edge preserving and noise smoothing
AF	27.376	0.1192	25.4278	174	Noise smoothing
UMF	27.380	1.0646	28.4076	3529	Edge sharpening
BF + UMF	27.374	0.3918	28.3763	1997	Edge sharpening
IASF	27.393	0.1192	29.1190	569	Edge sharpening and noise smoothing

**Table 3:** The comparison results for image data of target 2.

Objects	Average Intensity	STD		GMG	Characteristics
		Box D	Box E	Box E	
Original data	26.481	0.3840	12.0352	3362	-
WF	26.452	0.1772	11.4541	2902	Edge preserving and noise smoothing
BF	26.482	0.1833	11.9934	3376	Edge preserving and noise smoothing
AF	26.482	0.1772	9.9147	1716	Noise smoothing
UMF	26.483	1.2751	13.5684	7884	Edge sharpening
BF + UMF	26.483	0.5311	13.5174	7583	Edge sharpening
IASF	26.496	0.1776	13.8026	5904	Edge sharpening and noise smoothing

**Table 4:** The comparison results for image data of target 3.

Objects	Average Intensity	STD		GMG	Characteristics
		Box G	Box H	Box H	
Original data	49.203	1.4519	11.5198	3739	-
WF	49.146	1.2012	8.8813	1461	Edge preserving and noise smoothing
BF	49.195	1.2712	11.4804	3679	Edge preserving and noise smoothing
AF	49.204	1.2012	8.5635	1368	Noise smoothing
UMF	49.204	3.0548	13.5877	14,331	Edge sharpening
BF + UMF	49.197	1.7705	13.5777	13,740	Edge sharpening
IASF	49.297	1.2381	13.1734	6712	Edge sharpening and noise smoothing

**Table 5:** The comparison results of entropy for three different images.

Objects	Image of Target 1	Image of Target 2	Image of Target 3
Original data	4.0284	4.0663	4.8235
UMF	5.1530	5.2249	6.1282
BF + UMF	4.9597	5.0437	6.0132
IASF	4.8783	4.9323	5.8709

**Table 6:** Different filtering effects with different parameter values.

The Value of Parameter *k_p_*	The Value of Parameter *k_r_*	Filtering Effect	Note
*k_p_* = 0	*k_r_ ≥* 0	Low-pass, edge preserving, and noise smoothing	The bigger the value *k_r_*, the stronger noise smoothing
1 *> k_p_ >* 0	*k_r_* = 0	Weak edge enhancing	-
	*k_r_ >* 0	Weak edge enhancing and noise smoothing	The bigger the value *k_r_*, the stronger noise smoothing
	$k_{r}\gg \frac{\sigma_{I}^{2}}{\sigma_{r}^{2}}$	Noise smoothing	
*k_p_* ≥ 1	*k_r_* = 0	Edge enhancing	-
	*k_r_ >* 0	Edge enhancing and noise smoothing	The bigger the value *k_r_*, the stronger noise smoothing
	$k_{r}\gg \frac{\sigma_{I}^{2}}{\sigma_{r}^{2}}$	Noise smoothing	

## Data Availability

All data will be made available upon request to the corresponding author’s email with appropriate justification.
